# Imaging in Breast Cancer: Use of Magnetic Resonance Spectroscopy

**DOI:** 10.7759/cureus.9734

**Published:** 2020-08-14

**Authors:** Muhammad Ahmad Bilal Ahmadani, Shaista Bhatty, Zen ul Abideen, Maher Sohail Yaseen, Talha Laique, Jahanzeb Malik

**Affiliations:** 1 Radiology, Cardiac Centre, Bahawalpur, PAK; 2 Surgical Oncology, Ansari Surgical and Laproscopic Centre, Bahawalpur, PAK; 3 Physiology, D.G. Khan Medical College, Dera Ghazi Khan, PAK; 4 Pharmacology, Lahore Medical and Dental College, Lahore, PAK; 5 Cardiology, Rawalpindi Institute of Cardiology, Rawalpindi, PAK

**Keywords:** magnetic resonance spectroscopy, breast lesions and imaging modality, mri, magnetic resonance imaging, breast cancer, womens health

## Abstract

Background: Magnetic resonance spectroscopy (MRS) is used nowadays with increased specificity to distinguish between malignant and benign breast lesions.

Objective: To determine the diagnostic accuracy of MRS in malignant breast lesions.

Methodology: Newly diagnosed patients (n=158) having breast lesions diagnosed on ultrasound and mammography were enrolled to conduct the present study at Bahawal Victoria Hospital, Bahawalpur, Pakistan for six months. Enrolled patients were informed and consent was taken. Every patient underwent proton MRS using a 1.5 Tesla MR system. Fast scans in various planes were obtained. Mean ± standard deviation (SD) was given for age, size of the lump, and duration of the disease whereas frequency and percentage were given for benign and malignant breast lesions by SPSS version 26. A significant p-value was ≤0.05.

Results: The mean age of patients was 41.27 ± 5.48 years. The diagnosis of malignant breast lesions in 80 (50.63%) patients was shown by MRS whereas histopathology showed malignancy in 83 (52.53%) cases.

Conclusion: MRS is an accurate diagnostic modality for malignant breast lesions.

## Introduction

Breast cancer affects one in every nine Pakistani women [1]. Any breast lump raises the suspicion of malignancy among females of any age [1]. The majority of breast lesions are usually benign. Newly detected breast lesions are evaluated to see malignancy [2]. Effective screening with the help of breast self-examination, mammography, and clinical examination, can be beneficial in early diagnosis and treatment thereby decreasing mortality and morbidity for this disease [3-4].

If a lesion is malignant the imaging modalities can define its extent and find nonpalpable masses. These findings can alter the therapeutic approach. Mammography and ultrasonography are commonly used modalities for detecting breast cancer nowadays [5-6]. They have limitations in diagnosing early malignant lesions. This leads to the biopsy of the breast tissue but its sensitivity is 10% [7-8]. Magnetic resonance spectroscopy (MRS) of the breast is carried out as an investigational tool just to make a correct diagnosis of breast malignancies [9-11]. There is an elevated incidence of breast cancer among the female population but the data available is limited regarding the diagnosed malignant breast lesions, so we conducted the study to assess the accuracy of MRS as a diagnostic modality in breast cancer. It is relatively cheap, safe, and noninvasive as compared to histopathology which is reliable but invasive.

## Materials and methods

This was a descriptive, cross-sectional study. Newly diagnosed patients (n=158) having breast lesions diagnosed on ultrasound and/or mammography were enrolled at our institute following the approval by the Hospital’s Ethical Committee from January 2020 to June 2020. Enrolled patients were informed and written consent was taken. Every patient underwent MRS using a 1.5 Tesla MR system. Fast scans in various planes were obtained. The sample size of 158 cases was calculated with a 95% confidence level, a 7% margin of error, and taking an expected percentage of breast cancer as 65%. Enrolled females (30-50 years of age) presented with breast lesions with a duration of less than three months. Exclusion criteria involved patients who were unable to give informed consent, any second malignancy or metastasis, and pregnancy.

Mean ± standard deviation (SD) was given for age, size of the lump, and duration of the disease whereas frequency and percentage were given for benign and malignant breast lesions by SPSS version 26. Effect modifiers like age was controlled by stratification. Chi-square test was used to see their effects on outcomes considering p-value (≤ 0.05) as significant.

## Results

Among 158 enrolled patients, age ranged from 30 to 50 years. Demographic parameter like age was presented as mean ± SD (41.27 ± 5.48). The number of patients in different age ranges are summarized in Table 1.

**Table 1:** Age parameter among enrolled patients as Mean ± SD. SD, standard deviation

Age (Year)	Frequency	Percentage (%)
31-40	68	43.04
41-50	90	56.96
Total	158	100.0
Mean ± SD	41.27 ± 5.48	

The duration of the disease was presented as mean ± SD in Table 2.

**Table 2:** Duration of the disease among enrolled patients as Mean ± SD. SD, standard deviation

Duration of the disease (months)	Frequency	Percentage (%)
≤ 1 month	76	48.10
>1 month	82	51.90
Total	158	100.0
Mean ± SD	1.23 ± 0.85 months	

The demographic parameter of the breast lump size was presented as mean ± SD in Table 3.

**Table 3:** Size of the breast lump among enrolled patients as Mean ± SD. SD, standard deviation

Size of lump	Frequency	Percentage (%)
≤ 5 cm	93	58.86
>5 cm	65	41.14
Total	158	100.0
Mean ± SD	4.83 ± 2.35 cm	

Out of 158 participants, 74 patients were positive for breast cancer whereas 69 patients were negative on both MRS and histopathology as shown in Table 4.

**Table 4:** Finding of MRS and histopathology. MRS, magnetic resonance spectroscopy

Histopathology^++^	MRS Positive	MRS Negative	p-value
	74	09	0.736
Histopathology--	06	69	

MRS showed sensitivity of 90.62% and specificity of 94.44% in breast cancer patients. 

## Discussion

Magnetic resonance spectroscopy is a type of MRI that informs biochemically about tissue metabolism. Its diagnostic strength lies in the detection of markers of cancerous tissues (choline-containing compounds). Many previous studies have shown that usually, malignant breast lesions contain phosphocholine, which resonates at a chemical shift of 3.2 ppm [12]. MRS eliminates biopsy for benign lesions by showing the lack of a choline resonance at a chemical shift of 3.2 ppm. It takes less than 10 minutes for breast examination and thus accepted by patients if effective [13]. 

Its role in the early detection of malignant breast carcinoma is signified by the fact that its sensitivity is more than 90% for malignant breast lesions even before they can be palpated. Its radiation may be harmful to the patient. Nevertheless, its beneficiary effects outweigh the risks and inconvenience. Radiological techniques on various tissues and bones like spine, hip joints may be done to see evidence of metastasis of carcinoma [14]. 

It is a preferred examination for malignant breast cancer in females over 40 years. This age group has the highest prevalence of breast cancer due to hormonal fluctuations. Various studies have suggested that it is helpful even for the elderly women [15]. Our work is in line with the previous studies which suggest to start screening for breast carcinoma by mammography above 35 years of age. One study showed the sensitivity (89.5% and 83%) and specificity (92.3% and 85%) of MRS in the differentiation of malignant from benign breast lesions respectively [16]. It was demonstrated in our study with the sensitivity and specificity of 90.62% and 94.44%, respectively.

Our study had a couple of limitations. It was a single-center study with a small sample size. We did not perform a genetic study to see genetic variability among enrolled subjects. Large multi-center randomized controlled trials are needed to further ascertain the use of MRS in the early detection of breast cancer.

## Conclusions

Magnetic resonance spectroscopy is a relatively accurate modality for diagnosing breast lesions and has dramatically improved our ability to diagnose breast cancer in patients without any palpable masses. It can be used as a screening tool for at-risk age groups and in general oncology practice for an improved detection rate and early aggressive treatment.

## References

[1] Kumar A, Vohra LS, Bhargava S, Reddy PS (1999). Investigation of breast lumps: an evaluation. Med J Armed Forces India.

[2] Gotzsche PC, Nielsen M (2011). Screening for breast cancer with mammography. Cochr Datab Syst Rev.

[3] Sohail S, Alam SN (2007). Breast cancer in Pakistan - awareness and early detection. J Coll Phys Surg Pak.

[4] Siegel R, Naishadham D, Jemal A (2012). Cancer statistics, 2012. CA Cancer J Clin.

[5] Berg WA, Zhang Z, Lehrer D (2012). Detection of breast cancer with addition of annual screening ultrasound or a single screening MRI to mammography in women with elevated breast cancer risk. JAMA.

[6] Devolli-Disha E, Manxhuka-Kerliu S, Ymeri H, Kutllovci A (2009). Comparative accuracy of mammography and ultrasound in women with breast symptoms according to age and breast density. Bosnian J Basic Med Sci.

[7] Takhellambam YS, Lourembam SS, Sapam OS, Kshetrimayum RS, Ningthoujam BS, Khan T (2013). Comparison of ultrasonography and fine needle aspiration cytology in the diagnosis of malignant breast lesions. J Clin Diagn Res.

[8] Baek HM (2012). Diagnostic value of breast proton magnetic resonance spectroscopy at 1.5 T in different histopathological types. Sci World J.

[9] Shafqat G, Masror I, Rehan M, Afzal S (2011). Dynamic contrast enhanced MRI breast for lesion detection and characterization with histopathological co relation: preliminary experience at tertiary care hospital. J Pak Med Assoc.

[10] Begley JKP, Redpath TW, Bolan PJ, Gilbert FJ (2012). In vivo proton magnetic resonance spectroscopy of breast cancer: a review of the literature. Breast Cancer Res.

[11] Sardanelli F, Fausto A, Di Leo G, de Nijs R, Vorbuchner M, Podo F (2009). In vivo proton MR spectroscopy of the breast using the total choline peak integral as a marker of malignancy. Am J Roentgenol.

[12] Kvistad KA, Bakken IJ, Gribbestad IS (1999). Characterization of neoplastic and normal human breast tissues with in vivo 1H MR spectroscopy. J Magn Reson Imaging.

[13] Jagannathan NR, Kumar M, Seenu V (2001). Evaluation of total choline from in-vivo volume localized proton MR spectroscopy and its response to neoadjuvant chemotherapy in locally advanced breast cancer. Br J Cancer.

[14] Wasif N, Garreau J, Terando A, Kirsch D, Mund DF, Giuliano AE (2009). MRI versus ultrasonography and mammography for preoperative assessment of breast cancer. Am Surg.

[15] Badgwell BD, Giordano SH, Duan ZZ (2008). Mammography before diagnosis among women age 80 years and older with breast cancer. J Clin Oncol.

[16] Katz-Brull R, Lavin PT, Lenkinski RE (2002). Clinical utility of proton magnetic resonance spectroscopy in characterizing breast lesions. J Natl Cancer Inst.

